# Supplementary material for: Osteopathic practice in the United Kingdom: A retrospective analysis of practice data
Source: PLoS One. 2022 Jul 6;17(7):e0270806. doi: 10.1371/journal.pone.0270806 (PMC9258824; doi:10.1371/journal.pone.0270806)
Supplement: S1 File — (DOCX) [file pone.0270806.s001.docx]

Questionnaire survey content

| Part A provided full information about the survey and obtained consent.  Part B included questions about:   - - - Osteopath’s gender, age, and nationality     - Number of years of experience as an osteopath after initial graduation     - Practicing status     - Academic qualifications     - Working status as an osteopath (i.e. independent, employee)     - Location of practice (urban or rural)     - Practice environment (i.e. private practice, group practice with other osteopaths, interdisciplinary practice, clinic, hospital)   Part C included questions about:   - Description of patient and underlying condition:   - Month of encounter for selected patient   - Patient’s age   - Patient’s gender   - Primary reason for consultation   - Co-existing known conditions   - Duration of actual episode   - Type of onset of symptoms   - Localisation of symptoms   - Impact of symptoms on daily life   - Prior care for actual episode   - Any health professional who referred the patient - Description of first encounter:   - Informed consent for examination and treatment   - Treatment techniques employed during first encounter   - Use of adjunct therapies   - Recommendation of self-management strategies   - Duration of first encounter - Subsequent encounters:   - Number of encounters for primary reason for consultation   - Number of subsequent treatments (including adjunct therapies and self-management strategies)   - If applicable, description of referral to other health professional |
| --- |
