# Supplementary material for: Osteopathic practice in the United Kingdom: A retrospective analysis of practice data
Source: PLoS One. 2022 Jul 6;17(7):e0270806. doi: 10.1371/journal.pone.0270806 (PMC9258824; doi:10.1371/journal.pone.0270806)
Supplement: Attachment — Submitted filename: response-to-reviewers.docx [file pone.0270806.s003.docx]

**Response to reviewers**

Many thanks for all comments received from both reviewers. Below are our responses to comments received in your email of 14^th^ September 2021. Our resubmitted draft addresses the comments that were embedded within your file “PONE-D-20-30382_review (1).pdf”. Changes are in our resubmitted document, highlighted for visibility.

At the end of this document we also provide responses to comments and questions that were general in nature, or were raised by both reviewers, or where it was not clear which reviewer had raised the issue.

**Responses to comments received in email**

Reviewer 1:

- “In the results section, it would be really more interesting to have a comparison between the 2019 and 2009 results”.

There were some differences in the data collection methods that made us feel it more appropriate to comment on changes in the discussion rather than the results. For example: the 2009 study was prospective while the 2019 study was retrospective; we introduced some extra questions; and we asked participants for fewer patient records (10 in 2009, 8 in 2019). Due to these differences in data collection and changes to the questionnaire, we felt that a narrative discussion was a more appropriate way to highlight findings.

- “The discussion deserves to be reoriented to give less numbers and more explanation of changing trends and potential causes. The conclusions are also to be reoriented in relation to the results.”

We have added text to provide further context to both the discussion and the conclusion. In the discussion, we have added a paragraph indicating the value of standardised data collection, and how its relative paucity hinders the ability to identify trends and causes, particularly in comparison to other professions. Our findings indicate few changes within the UK’s osteopathic profession since 2009, although where changes have been observed these are described on page 18 of the current draft. We have restructured the paper’s conclusion to highlight these points and to follow more clearly from the results and discussion.

- “The conclusion in the abstract is not related directly to results presented”

A sentence has been added to the conclusion in the abstract for clarification.

- “What changes have been made to the original [survey] tool? Could this change the content validity?”

Text has been added regarding the changes to the survey instrument and its validity in page 5 in the section titled “the questionnaire survey”.

- “Table 2 could be shorter and mention that some approaches have not been reported by any osteopath”
- “Standardize references including surveys previously conducted in the UK”

These have been addressed in the latest submission; the table has been shortened as suggested and the references have been revised and standardised throughout.

- “Not sure if it is relevant to repeat the statistics at the beginning of the discussion. Maybe just the broad findings”.

This section has been rephrased and moved out of the Discussion and into the Results section.

- “The comparison with other countries is very similar to the discussion in the systematic review. Possibility to put more emphasis on what is different between the current study and the systematic review cited”

We have expanded the discussion to clarify where data is compared with other countries, and to describe difficulties in comparing osteopathic data with that from other professions. Please refer to our preceding responses regarding comparison with 2009 data.

- “What might be the implication of under-representation of certain age or gender groups?”

We have provided a brief explanation about why we feel any under-representation is unlikely to have produced significant biases within the data, in the “strengths and limitations”.

- “The conclusion is more of a perspective than a conclusion related to the results or evolution of the osteopaths' practice.”

The conclusion has been rewritten to highlight the key changes in the UK osteopathic profession since the 2009 study. Additionally, further references have been added (Moore, Bryant & Olivier 2012; Clar *et al.* 2014) to draw attention to the need for standardised data collection not only for a better understanding of the evolution of osteopathy, but also of related professions within the UK and internationally.

Reviewer 2:

- “[T]he major considerations centre on how the results of the study are contextualised to the wider literature - the reader is left to ponder the value of the data in the study.”
- “At present, the Discussion is largely a descriptive comparison to 2009 work with little reference to other works to explain why changes were observed or not observed. [...] greater attention should be given to discussing the results in the context of other literature.”

As per our preceding comments, these issues have been addressed in the latest submission.

Journal requirements:

- “Please include additional information regarding the survey or questionnaire used in the study and ensure that you have provided sufficient details that others could replicate the analyses. […] In your Data Availability statement, you have not specified where the minimal data set underlying the results described in your manuscript can be found”

A minimal set of the data underlying the results has been made, containing data that is not in the paper itself. This is available online at <https://www.ncor.org.uk/osteosurvey-minimal-dataset/> and in a permament public registry at <https://doi.org/10.17605/OSF.IO/A25XW>

The UK’s National Council for Osteopathic Research (NCOR) is an independent charity. Professor Carnes is no longer employed by NCOR. The fully-anonymised data set will be available on request to NCOR by emailing [info@ncor.org.uk](mailto:info@ncor.org.uk) The full questionnaire runs to 213 pages, and again can be available on request by contacting NCOR.

- “Please ensure that you refer to Figures 1 and 2 in your text as, if accepted, production will need this reference to link the reader to the figure.”
- “We note you have included a table to which you do not refer in the text of your manuscript. Please ensure that you refer to Tables 1,2 and 3 in your text.”

These have been addressed in the current draft.

- Competing interests and AP’s position as a “Director at the Institute of Osteopathy, the members' organisation for the UK's osteopathic profession”.

We confirm that this does not alter our adherence to PLOS ONE policies on sharing data and materials, and does not influence the sharing of data and/or materials.

**Responses to other comments**

- “[P]lease ensure that the dates for data collection are consistent and clearly described.”

We believe these years are now used consistently and accurately throughout. The survey was conducted between July 2019 and January 2020, and asked participants to review their patient notes and practice activities from the year 2018.

- “[S]hould there be other strategies to increase access to osteopathic care?”

Factors surrounding the access to osteopathic care have social and economic implications that we feel are beyond the scope of this paper. While certainly a worthwhile topic for discussion, the full implications of such issues would encompass not just the osteopathic profession but the NHS and UK Government healthcare and social policies, and private insurance. The aim of our study was to describe the osteopathic patient profile and the care they receive.

Many thanks to all for their comments received, and we welcome any further requests for clarification or correction.
