# Supplementary material for: Osteopathic practice in the United Kingdom: A retrospective analysis of practice data
Source: PLoS One. 2022 Jul 6;17(7):e0270806. doi: 10.1371/journal.pone.0270806 (PMC9258824; doi:10.1371/journal.pone.0270806)
Supplement: Attachment — Submitted filename: plosone-response-to-reviewer-final.docx [file pone.0270806.s004.docx]

Dear Editor,

In response to reviewer comments:

*I mean that you rewrite the Discussion giving meaningful sentences consistent with the data and the findings.
I do not mean "move the overall summary of findings out of the Results and back into the Discussion". Revision by the authors is OK .
Thanks and regards
Dr Bhamidipati Narasimha Murthy*

We are at a bit of a loss as to how to respond to Dr Murthy. In each paragraph in the discussion we cite the data to support each point made in the discussion so that our comments are fully underpinned by our survey results. We believe the points we highlight are meaningful and will help the osteopathic profession reflect on their position in the health care sector. We have placed our current findings in comparison with the earlier national data collection in osteopathy in 2009 and believe this context is most relevant to the discussion. In our discussion, we have also placed the current data within the context of practice in European countries where such data exists. What we cannot do is further interpret the findings as the interpretation will vary dependent on the reader and their standpoint (clinician, commissioner, professional body or regulator, for example).

Below we summarise the point made in each paragraph to illustrate the meaning of our findings. We have adjusted some of the text in the manuscripts to expand or highlight the points made.

**Discussion**

Paragraph 1: We comment that online data capture had a similar response rate to paper-based survey 10 years on.

Paragraph 2: We highlight the similarities between the patient presenting complaints in 2009 and 2018. And comment that little has changed.

Paragraph 3: We make the point that survey findings indicate osteopaths deliver a package of care in line with UK NICE guidance for the management of low back pain.

Paragraph 4: We make 2 points: access to treatment still remains limited by ability to pay; and that UK osteopaths still work predominantly as first contact practitioners in the private sector.

Paragraph 5: We make the point that the data suggests an increase in consultations for much younger children.

Paragraph 6: We point out that the relationship between private and publicly funded care systems are closely linked for patients seeking osteopathic care.

Paragraph 7: We report that the data indicates a greater diversity of care offered in 2018 and /or an increase in demand for non-musculoskeletal care.

Paragraph 8: We comment on the change in proportion of osteopaths serving the UK population.

Paragraph 9: We note the patient population seeking care have more persistent conditions reflecting the aging UK demographic who have more long-term conditions.

Paragraph 11: We cite similarities and differences of the UK with other countries.

Paragraph 12: We compare the UK osteopathic profession with other allied health professions.

Paragraphs 13 ,14 and 15: We discuss the strengths and limitations of the research.

Paragraph 16 and 17: We consider other research especially the need for PROMs and PREMs.

Paragraph 18: We make comments about what the data means in terms of health service delivery in the UK.

Paragraph 19 is the conclusion.

We look forward to your Editorial Team’s response.

With best wishes,

Austin Plunkett (lead author)

Dawn Carnes

Carol Fawkes
